# Sensory Perception in Lumbosacral Radiculopathy with Radicular Pain: Feasibility Study of Multimodal Bedside-Suitable Somatosensory Testing

**DOI:** 10.15388/Amed.2021.28.1.18

**Published:** 2021-04-29

**Authors:** Alfredas Vaitkus, Jūratė Šipylaitė

**Affiliations:** Clinic of Anaesthesiology and Intensive Care, Institute of Clinical Medicine, Faculty of Medicine, Vilnius University, Vilnius, Lithuania; Centre of Anaesthesiology, Intensive Therapy and Pain Management, Vilnius University Hospital Santaros Klinikos, Vilnius, Lithuania ORCID: https://orcid.org/0000-0001-5869-7726; Clinic of Anaesthesiology and Intensive Care, Institute of Clinical Medicine, Faculty of Medicine, Vilnius University, Vilnius, Lithuania; Centre of Anaesthesiology, Intensive Therapy and Pain Management, Vilnius University Hospital Santaros Klinikos, Vilnius, Lithuania ORCID: https://orcid.org/0000-0001-7099-1741

**Keywords:** sensory testing, bedside testing, sensory phenotyping, back pain, lumbosacral radiculopathy

## Abstract

**Background:**

Somatosensory testing could be useful in stratifying pain patients and improving pain treatment guidelines. Bedside-suitable techniques are searched for application in daily clinical practice. This study aimed to characterize chronic unilateral lumbosacral radiculopathy (LSR) patients with radicular pain using multimodal bedside-suitable somatosensory testing.

**Materials and methods:**

We evaluated 50 chronic unilateral LSR patients with radicular pain (LSR group) and 24 controls (Control group). Sensory testing was performed using a battery of bedside sensory tests (10g monofilament, 200–400 mN brush, Lindblom rollers with controlled 25°C and 40°C temperature, and 40g neurological pin and investigator’s finger pressure). Participants had to rate their sensory perceptions on both legs at multiple test points within L3 to S2 dermatomes. Characteristics of the testing process and sensory disturbances were analyzed.

**Results:**

LSR group showed sensory disturbances in 82% of patients. The Control group showed no sensory disturbances. Sensory testing took longer (*p* < 0.001) in the LSR group (29.3 ± 6.5 minutes per patient) than in the Control group (20.5 ± 5.2). Nine sensory phenotypes were detected in the LSR group according to individual sensory disturbances within 5 superficial tests.

**Conclusions:**

The applied multimodal bedside-suitable somatosensory testing battery is suitable for sensory evaluation and characterization of LSR patients. Grouping of allied sensory phenotypes revealed some tendencies in pain intensity characteristics.

## Abstract (lt)

**Jutimų suvokimas sergant juosmens ir kryžmens radikulopatija su šakneliniu skausmu: multimodalinio somatosensorinio ištyrimo prie ligonio lovos tinkamumo tyrimas:**

**Santrauka.***** Įvadas.*** Somatosensoriniai tyrimai galėtų būti naudingi klasifikuojant skausmą patiriančius pacientus ir gerinant jų gydymo gaires. Ieškoma jutimų tyrimo metodikų, tinkamų atlikti prie ligonio lovos ir pritaikomų kasdienėje klinikinėje praktikoje. Šiuo tyrimu buvo siekiama įvertinti pacientų, sergančių lėtine vienpuse juosmens ir kryžmens radikulopatija su šakneliniu skausmu, jutimų sutrikimus atliekant multimodalinį somatosensorinį ištyrimą prie ligonio lovos.

***Priemonės ir metodai.*** Mes ištyrėme 50 lėtine vienpuse juosmens ir kryžmens radikulopatija su šakneliniu skausmu sergančių pacientų ir 24 kontrolinės grupės tiriamuosius. Jutimų tyrimas buvo atliktas naudojant prie ligonio lovos tinkamų sensorinių testų bateriją (10 g monofilamentą, 200–400 mN šepetėlį, kontroliuojamos 25 °C ir 40 °C temperatūros Lindblomo volelius, 40 g neurologinę adatėlę bei tyrėjo piršto paspaudimą). Tyrimo dalyviai turėjo įvertinti sensorinių dirgiklių sukeliamus pojūčius abiejų kojų simetriniuose taškuose išilgai L3–S2 dermatomų. Atlikta jutimų tyrimo eigos ir nustatytų jutimų sutrikimų analizė.

***Rezultatai.*** Juosmens ir kryžmens radikulopatijos grupės 82 proc. tiriamųjų buvo nustatyti jutimų sutrikimai. Kontrolinės grupės tiriamiesiems jutimo sutrikimų nenustatyta. Vidutinė vieno paciento jutimų tyrimo trukmė visa sensorinių testų baterija juosmens ir kryžmens radikulopatijos grupėje buvo ilgesnė nei kontrolinėje grupėje (29,3 ± 6,5 min vs 20,5 ± 5,2 min; p < 0,001). Juosmens ir kryžmens radikulopatijos grupės tiriamiesiems pagal individualius jutimo sutrikimus penkiuose paviršinių jutimų testuose buvo nustatyti devyni sensoriniai fenotipai.

***Išvados.*** Tyrime naudota multimodalinio somatosensorinio ištyrimo prie ligonio lovos testų baterija yra tinkama jutimams vertinti. Grupuojant giminingus sensorinius fenotipus buvo nustatyta skausmo intensyvumo skirtumų.

**Raktiniai žodžiai:** sensorinis tyrimas, ištyrimas prie ligonio lovos, sensorinis fenotipavimas, nugaros skausmas, juosmens ir kryžmens radikulopatija.

## Introduction

Low back pain (LBP) is a leading cause of disability defined by the global all-age years lived with disability (YLDs) index in 2017 [1]. Classic LBP treatments vary from passive to active, from noninterventional to surgical, and from traditional to modern. Still, the question of which method suits each patient best remains open, as evidence is often insufficient to conclude [2]. Treatment response varies significantly among all treatment methods [3]. There is a tendency of phenotyping pain patients to characterize them more accurately and facilitate the selection of available treatments [3]. Quantitative sensory testing (QST) is used to evaluate sensory disturbances, and it is applicable in patients with back pain pathology [4]. Standardized and automated QST is a reliable method supplying detailed characteristics of sensory function. However, its application in daily clinical practice remains limited due to the expensiveness of equipment, considerable consumption of time, and the need for personnel training.

Also, for the application of comprehensive QST in patients with radiculopathy, there is uncertainty regarding site selection for sensory testing. Low back pain with radiculopathy could manifest with the pain of different mechanisms – nociceptive, referred, neuropathic, or a mix of any of these. Pain is usually related to patients’ posture, activity, and some other factors [5]. Therefore, it is difficult to determine the origin and mechanisms at patients’ reported pain sites. QST protocols recommend testing the site of greatest pain [6]. Radicular pain related to lumbosacral radiculopathy (LSR) is often reported along the involved segments or at separate locations of its. If to test several sites using comprehensive QST protocol, it would take plenty of time. This would question its accuracy due to the patient’s fatigue. Therefore, it would be appropriate to screen all LSR related segmental zones in a reasonable amount of time. More simple nonautomated QST approaches and sensory mapping techniques have also been suggested to address this problem [7]. Furthermore, the qualitative sensory testing principle was proposed and tested for the detection of atypical odontalgia [8], stenosis involving the L5 nerve root [9], and for assessment of nerve block quality with local anesthetic [10].

We hypothesized that a bedside-suitable sensory testing battery describing qualitative characteristics could demonstrate the variability of sensory disturbances in LSR patients with radicular pain and could be applicable for sensory phenotyping.

## Methods

### Study aims and objectives

The presented study is a prospective diagnostic study aimed to explore the feasibility of bedside-suitable multimodal somatosensory testing for the evaluation of LSR patients with radicular pain. The objectives of this study were: to assess the capability of selected testing battery to detect sensory disturbances in LSR patients, also to evaluated the time needed to perform sensory testing and patient cooperation issues. The secondary aim of this study was to characterize typical sensory disturbances in LSR patients with radicular pain. The main study group was collected of chronic painful unilateral LSR patients (LSR group). The Control group consisted of chronic pain patients without LSR but with pain syndromes anatomically unrelated to the body region being explored by sensory testing, i.e., pain syndromes not involving the lower waist and legs. The decision to form the Control group of pain patients (not healthy subjects) was made because sensory testing is a psychophysical testing type. Therefore, subjects free of pain would have a different psychophysical background.

### Ethics

The Vilnius Regional Biomedical Research Ethics Committee approved this study protocol (#158200-15-772-289). All participants were informed about the goal and the study process and gave their informed consent to participate.

### Participants

The study was carried out from 2015 to 2018. The patients were recruited from those who were referred to the Pain Medicine Centre at the Vilnius University Hospital Santaros Klinikos (Vilnius, Lithuania) on a scheduled basis. During the screening, all consecutive patients meeting inclusion criteria for the LSR or Control group were invited.

The inclusion criteria for the LSR group were chronic painful unilateral LSR patients complaining of leg pain (with or without low back pain) and pain duration of >3 months. Criteria for painful LSR were typical radiating segmental pain with at least one sign of neurological impairment of corresponding spinal segments (positive straight leg raise (SLR) test at an interval of 0–70° or sensory impairment at testing with a standard neurological pin or motor function impairment less than 5th-grade muscle strength according to Lovett’s scale or impairment of deep tendon reflexes). The results of the radiological examination had to be compatible with clinical symptoms.

The Control group was formed of chronic pain patients (pain duration of >3 months) without LSR and with pain syndromes anatomically unrelated to the lower waist and legs. The age limits for both groups were 18 to 85 years.

Exclusion criteria for both groups are presented in Table 1.

**Table 1.** Exclusion criteria.

**1. Exclusion criteria for both LSR and control groups:** patients with diseases or states capable of interfering with sensory evaluation (e.g., osteoarthritis or osteoarthrosis of the leg joints; systemic inflammatory diseases; local or systemic infection; renal or hepatic insufficiency; anemia; pregnancy or breastfeeding; oncological disease; diabetes mellitus or any other endocrine disease; polyneuropathy or mononeuropathy; previous chemotherapy, radiation, trauma, polio, stroke, CNS or spinal surgery, or surgery in the regions of sensory testing) inability to understand study instructions or documentation properly patients not able to tolerate supine position required for performing sensory testing
**2. Additional exclusion criteria for the LSR group:** complicated lumbosacral radiculopathy with cauda equina syndrome received epidural steroid injections less than 6 months before study initiation any other localized pain stronger than 3 points on NRS 0-10 any pain on the opposite to the affected by radiculopathy leg patients in which a detected sensory disturbance was not consistent with LSR

LSR – lumbosacral radiculopathy; CNS – central nervous system; NRS – numeric rating scale

Pain evaluation consisted of: pain intensity evaluated separately for low back and leg (Brief Pain Inventory short form (BPI-SF) Pain items, numeric rating scale 0 to 10 points (NRS 0-10)), pain impact on functioning (BPI-SF Interference items, NRS 0-10), anxiety and depression evaluation (Hospital Anxiety and Depression Scale (HADS) score), screening for neuropathic pain component (painDETECT Questionnaire (PD-Q) score). Assessment of magnetic resonance imaging (MRI) and/or computed tomography (CT) of the lumbosacral spine not older than 6 months were collected and analyzed. Sensory testing was performed on all patients, collecting data on sensory disturbances, testing duration, and any test completion problems.

### Sensory testing

A single investigator performed sensory testing. Dermatomes L3, L4, L5, S1, and S2, according to the dermatomal distribution map from *Bonica’s Management of Pain* [11] were tested at three levels: thigh, calf, and foot. At least 2 points for each level of every dermatome were tested. Hence, at least 6 points for 1 dermatome were tested in one leg, starting proximally and proceeding distally. In every modality each dermatome was tested sequentially one by one. Similar sensory testing was performed in both groups. Sensory function was evaluated by comparing sensory perceptions on both legs. In the LSR group, the involved (painful) leg was compared to the nonpainful leg. Patients were asked to compare and report their sensory perceptions at each pair of tested symmetrical points on both legs (“mirror image” points) at least twice. The quality of sensory perception in every tested point was rated separately as normal sensitivity (if similar to control side), hyposensitivity (reduced sensory perception or no perception), or hypersensitivity (increased sensory perception or painful sensation in cases of nonpainful stimuli). When two tests at any test points were inconsistent, third or more tests were performed with a slight change in the testing point location and comparison to a secondary control point. The anterolateral same-side surface of the trunk at the umbilical level was used as a secondary control site. 

If the perception in the radiculopathy-affected leg was rated as being normally sensitive at all points examined, the tested modality was qualified as normal. If the sensory perception in any of the tested dermatomes was rated as hyposensitive or hypersensitive in at least two adjacent test points along the dermatome, then the tested modality was qualified as hyposensitive or hypersensitive, respectively. The dermatomes with sensory disturbances had to correspond to the segments defined in the clinical diagnosis of radiculopathy. Noncompliance with radiculopathy-affected segments was qualified as an exclusion criterion.

The full sensory testing battery consisted of 6 tests (5 superficial and 1 deep sensory perception), as listed in Table 2. All superficial sensory tests were performed using standardized instruments that are approved and manufactured for this purpose. 

**Table 2.** The battery of sensory tests used in the study with corresponding sensory modalities and axons tested. All tests are presented in the order of testing. The order of tests performed and general requirements for testing were followed according to the guidelines set by Walk et al. (2009) [7].

**Sensory modality**	**Axon type primarily tested [12]**	**Instruments and their specifications**
**Superficial sensory tests**		
Mechanical tactile nonpainful static	Aβ	10g monofilament (Neuropen, Owen Mumford Ltd, Woodstock, UK)
Mechanical tactile nonpainful dynamic	Aβ	200.400 mN brush (SENSELab Brush-05, Somedic SenseLab AB, Sosdala, Sweden)
Thermal of innocuous cold	Aδ	Lindblom roller with a controlled temperature of 25°C (Rolltemp, Somedic SenseLab AB, Sosdala, Sweden)
Thermal of innocuous warmth	C	Lindblom roller with a controlled temperature of 40°C (Rolltemp, Somedic SenseLab AB, Sosdala, Sweden)
Mechanical tactile painful, pinprick	Aδ, C	40g neurological pin (Neurotip/Neuropen, Owen Mumford Ltd, Woodstock, UK)
**Deep tissue sensory test**		
Deep pressure	A, C	Finger pressure with moderate force

### Statistics

We used descriptive statistics such as frequency tables and means (with standard deviation) to describe quantitative and qualitative data, respectively. The normality of the distribution was assessed using the Kolmogorov–Smirnov test. Differences between the two independent quantitative normally distributed, nonnormally distributed, and qualitative groups were evaluated by the Student’s t-test, Mann–Whitney U test, and the chi-square test, respectively. A Kruskal–Wallis one-way ANOVA was used to assess the mean differences in more than 2 groups. The Bonferroni correction was used for pairwise comparisons. A two-tailed *p*-value less than 0.05 was considered significant. Statistical analysis was performed using the statistical analysis software (SAS) package version 9.2. 

## Results

### Study flow

**Figure 1.**
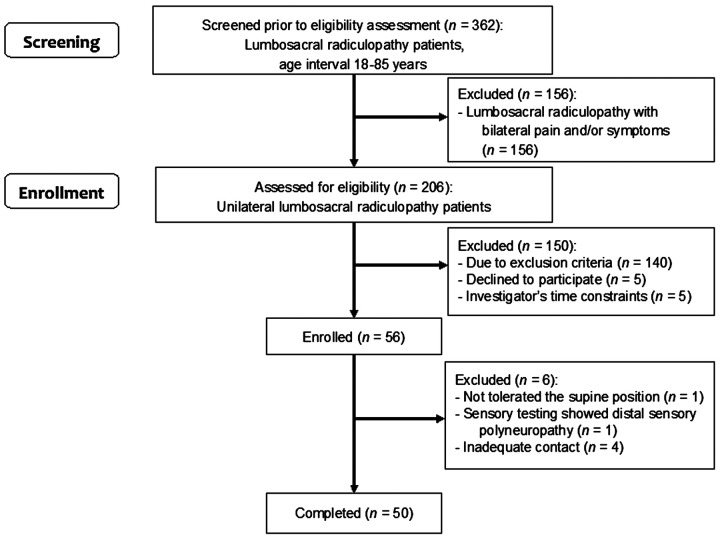
Flowchart for the inclusion of study participants into the LSR group.

A total of 362 patients were screened for enrollment into the LSR group. 56 patients were enrolled into the LSR group, and 50 of them completed it (Figure 1). Twenty-four patients were enrolled into the Control group and completed the study.

### General characteristics

Fifty LSR patients (30 women, 20 men; mean age ± SD: 59.5 ± 10.3, range 38–79 years) and 24 control group patients (17 women, 7 men; mean age ± SD: 56.1 ± 12.7, range 30–85 years) completed this study. Post hoc analysis showed both groups to be age- and sex-matched. The groups differed reliably in weight (83.0 vs 73.0 kg, *p* = 0.017) and body mass index (29.0 vs 26.1, *p* = 0.001), which is consistent with data confirming association of higher weight with LBP [13].

### Pain characteristics

Pain in the leg was present in all LSR patients (52% right side, 48% left side). Low back pain was present in 88% of the LSR patients. Detailed pain and related characteristics of the LSR group are presented in Table 3.

Other complaints concomitant to pain were reported: paresthesia/numbness – 72%, muscle cramps – 22%, feeling of cold in the leg – 12%, and leg swelling – 4%.

Pain characteristics in the Control group were: pain duration 43.6 ± 70.2 months, range 3–312, pain intensity as average during last week 5.3 ± 1.16 points on NRS 0-10, range 3–7. These variables differed from the LSR group with statistical significance of *p *= 0.029 and *p *= 0.023, respectively, but this was treated as indeterminant for comparative sensory testing results.

### Neurological evaluation

**Figure 2.**
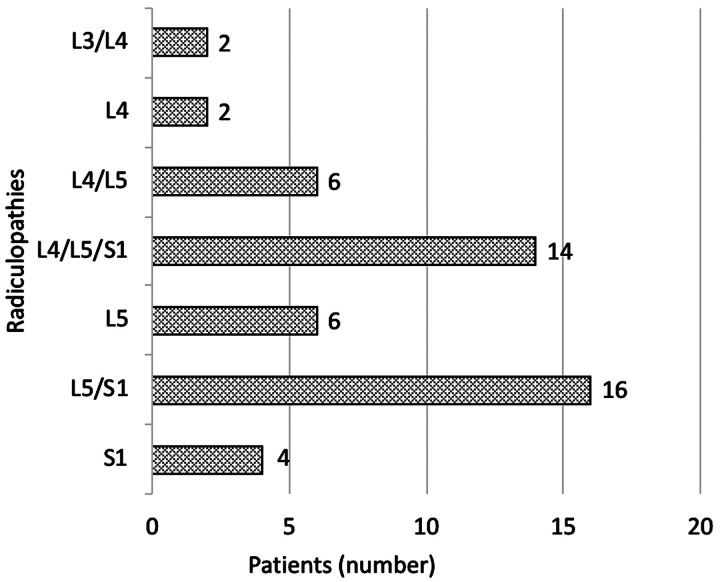
Nerve root impairment according to diagnosis in the LSR group (*n *= 50).

The distribution of nerve root impairment according to LSR diagnosis is shown in Figure 2. L5 root was the most often involved in the diagnosis, 80% of the cases, S1 in 68%, L4 in 48%, and L3 in 4% of cases.

**Table 3.** Pain and related characteristics in the LSR group (*n* = 50).

**Variable**	**Summary statistic** Mean (± SD), (min–max)
Pain duration – Low back (months):	
Overall	61 (± 61), 0–255
Current intensity	5 (± 5), 0–26
Pain duration – Leg (months):	
Overall	29 (± 34), 3–180
Current intensity	5 (± 4), 0–16
Pain intensity – Low back (BPI-SF, NRS 0-10):	
Worst during last week	7.0 (± 2.6), 0–10
Least during last week	2.0 (± 2.1), 0–6
Average during last week	4.9 (± 2.1), 0–8
Current pain	4.2 (± 2.8), 0–10
Pain intensity – Leg (BPI-SF, NRS 0-10):	
Worst during last week	7.4 (± 1.5), 4–10
Least during last week	1.8 (± 2.5), 0–8
Average during last week	5.3 (± 2.0), 2–10
Current pain	3.7 (± 3.2), 0–10
Impact on functioning (BPI-SF, NRS 0-10):	
General activity	7.5 (± 2.0), 2–10
Mood	7.3 (± 2.5), 0–10
Walking	7.5 (± 1.9), 2–10
Work	8.2 (± 1.3), 5–10
Relations	5.2 (± 3.2), 0–10
Sleep	6.1 (± 3.1), 0–10
Enjoyment of life	7.2 (± 2.7), 0–10
Mean of all	7.0 (± 1.8), 3.3–10
States of anxiety and depression (HADS score):	
Anxiety	8.0 (± 4.7), 1–18
Depression	5.8 (± 2.1), 1–11
Neuropathic pain component (PD-Q score)	9.5 (± 3.8), 2–20

LSR – lumbosacral radiculopathy; SD – standart deviation; BPI-SF – Brief Pain Inventory short form; NRS – numeric rating scale; HADS – Hospital Anxiety and Depression Scale; PD-Q – painDETECT Questionnaire.

Sensory impairment by testing with a standard neurological pin was detected in 76% of patients, impairment of deep tendon reflexes was detected in 64% of patients, and motor function impairment was detected in 72% of patients. Positive SLR test was detected in 88% of patients with 39° ± 16° (range 15° –70° ). Eight percent of patients without any neurologic deficit were diagnosed with LSR based on typical segmental radicular pain distribution with a positive SLR test present. Neurogenic claudication was reported by 38% of patients.

### Radiographic characteristics

The sole selective radiologic finding corresponding to spinal nerve involvement of LSR diagnosis was detected in 54% of cases. All other patients (46%) could be defined as having diffuse/multiple degenerative changes compatible with LSR diagnosis, though it is difficult to determine the exact anatomical site of spinal nerve impingement.

### Sensory characteristics

Sensory testing took significantly longer in the LSR group (29.3 ± 6.5 minutes per patient) than in the Control group (20.5 ± 5.2 minutes per patient), *p* < 0.001.

Patients from the Control group showed no sensory impairment or asymmetry between the left and right legs to all tested sensory modalities.

**Figure 3.**
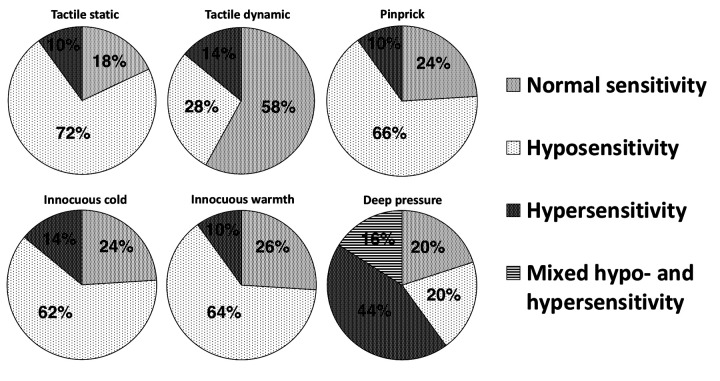
Proportions of patients with sensory disturbances detected at different modalities in the LSR group (*n* = 50).

In the LSR group, 82% of the patients showed sensory disturbances. The full spectrum of disturbances is presented in Figure 3. A single type of disturbance inside each separate sensory modality (hypersensitivity or hyposensitivity) was registered per patient except in deep pressure testing. In deep pressure testing, 16% of cases showed mixed hypo- and hypersensitivity (at different testing points).

The frequency of hyposensitivity to the tactile dynamic test was much rarer compared to other types of superficial sensory tests. Statistical comparison of proportions showed that the percentage frequency of hyposensitivity and other sensory types (normal sensitivity and hypersensitivity) differed significantly in tactile dynamic testing (28% and 72%) if to compare to tactile static testing (72% and 28%, *p* < 0.001), to pinprick testing (66% and 34%, *p* < 0.001), to cold testing (62% and 38%, *p* = 0.001), and to warmth testing (64% and 36%, *p* < 0.001).

Grouping LSR patients by disturbances in each superficial sensory test revealed nine sensory phenotypes (Table 4).

**Table 4.** Sensory phenotypes according to superficial sensory testing in the LSR group (*n* = 50).

**Phenotypes**	**Number of patients**	**Sensory disturbances^a^**					**Sensory group**
		**Tactile static**	**Tactile dynamic**	**Pinprick**	**Innocuous cold**	**Innocuous warmth**	
I	9	Normal	Normal	Normal	Normal	Normal	Normal sensitivity group
II	2	Hypo	Normal	Normal	Normal	Normal	Hyposensitivity to 1 to 3 modalities group
III	1	Hypo	Normal	Normal	Normal	Hypo	
IV	2	Hypo	Normal	Hypo	Hypo	Normal	
V	15	Hypo	Normal	Hypo	Hypo	Hypo	Hyposensitivity to 4 to 5 modalities group
VI	12	Hypo	Hypo	Hypo	Hypo	Hypo	
VII	2	Hypo	Hyper	Hypo	Hypo	Hypo	
VIII	2	Hypo	Hypo	Hypo	Hyper	Hypo	
IX	5	Hyper	Hyper	Hyper	Hyper	Hyper	Hypersensitivity group

^a^ Meanings: Normal – normal sensitivity, Hypo – hyposensitivity, Hyper – hypersensitivity.

The sensitivity of each superficial sensory test in the detection of LSR was as follows: tactile static test – 82%, pinprick – 76%, innocuous cold – 76%, innocuous warmth – 74%, and tactile dynamic – 42%. There were no superficial sensory test combinations that increased single tactile static testing sensitivity in detecting lumbosacral radiculopathy.

**Figure 4.**
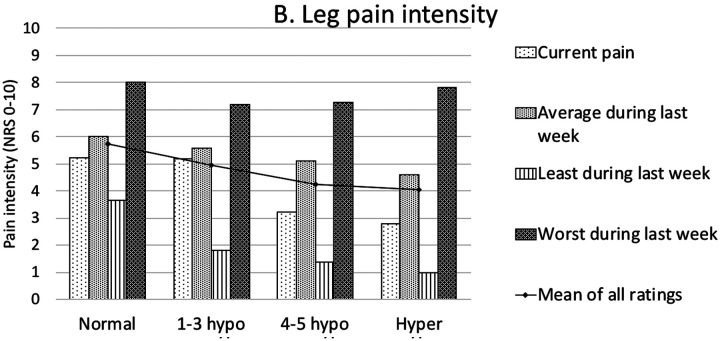
Low back (A) and leg (B) pain intensities differed between groups of sensory phenotypes.****Pain intensities are presented as means in points on NRS 0-10. Differences between groups are insignificant. NRS – numeric rating scale.

Correlations of sensory disturbances in the LSR group with other patient characteristics (demographic, pain, radiologic spine changes) have been tested. As patient groups in different phenotypes were small, the following grouping of allied phenotypes has been applied. The following groups were formed (Table 4): “Normal” (phenotype I patients with normal sensitivity in all tested modalities), “1-3 hypo ” (phenotype II–IV patients with relatively less hyposensitivity – in 1, 2, or 3 modalities), “4-5 hypo ” (phenotype V–VIII patients with relatively more hyposensitivity – in 4 or 5 modalities) and “Hyper ” (phenotype IX patients with hypersensitivity in all tested modalities). Though statistically significant differences were not detected, the “Normal ” patient group tended to show higher pain intensities than other groups in all low back and leg pain categories. The “Hyper ” patient group tended to show lower pain intensities in most pain categories (Figure 4). Larger patient groups are needed to determine whether correlations are statistically significant. A full list of distribution of characteristics across groups of sensory phenotypes is presented in Table 5.

### Patient cooperation

Thirteen (9.3%) of 140 excluded patients were categorized with “inability to understand study instructions or documentation properly. ” This group consisted of patients with insufficient cognitive function or language knowledge (a peculiarity of the regional multilingual population). Additionally, four (7.1%) of 56 patients enrolled in the trial were excluded due to inconsistent or inconclusive sensory testing.

## Discussion

Qualitative type sensory testing is relatively uncommon in clinical trials as comprehensive automated QST still reigns as the gold standard. In contrast to QST, qualitative sensory testing tools create a fixed-intensity stimulus. They allow classification of sensory perception as either normal or not if compared to an intact reference testing site without determining the degree of sensory dysfunction. Like QST, qualitative sensory testing provides information on the perception of stimulation to different types of receptors and their nerve fibers. 

**Table 5.** Analysis of characteristics between groups of sensory phenotypes in the LSR group. Differences between groups are insignificant.

Variable	Groups of sensory phenotypes			
	Normal^a^ (*n* = 9)	1-3 hypo^b^ (*n* = 5)	4-5 hypo^c^ (*n* = 31)	Hyper^d^ (*n* = 5)
Gender (n (%)):				
Female	6 (66.7)	3 (33.3)	4 (80.0)	1 (20.0)
Male	19 (61.3)	12 (38.7)	1 (20.0)	4 (80.0)
Age (years, mean (± SD))	67.6 (± 9.3)	54.4 (± 6.5)	58.8 (± 10.1)	55.0 (± 10.7)
Body mass index (kg/m2, mean (±SD))	30.2 (± 3.6)	27.4 (± 5.1)	29.0 (± 4.2)	28.0 (± 4.1)
Straight leg raise (degrees, mean (±SD))	49 (± 13)	48 (± 13)	35 (± 13)	34 (± 14)
Pain duration – Low back (months, mean (±SD)):				
Overall	50 (± 41)	48 (± 63)	65 (± 61)	68 (± 99)
Current intensity	3 (± 2)	3 (± 4)	6 (± 5)	7 (± 11)
Pain duration – Leg (months, mean (±SD)):				
Overall	31 (± 37)	37 (± 32)	28 (± 37)	20 (± 18)
Current intensity	3 (± 1)	3 (± 4)	5 (± 4)	5 (± 4)
Impact on functioning (BPI-SF Interference Items, NRS 0-10, mean (±SD))				
General activity	8.2 (± 1.4)	6.4 (± 4.0)	7.5 (± 1.6)	7.6 (± 2.3)
Mood	8.3 (± 1.5)	5.6 (± 3.4)	7.1 (± 2.5)	8.0 (± 1.9)
Walking	7.7 (± 1.6)	6.2 (± 3.5)	7.7 (± 1.5)	6.6 (± 2.2)
Work	8.4 (± 1.4)	8.6 (± 0.5)	8.0 (± 1.4)	8.2 (± 1.5)
Relations	4.4 (± 3.8)	7.0 (± 1.9)	5.2 (± 3.1)	4.4 (± 3.8)
Sleep	7.2 (± 1.8)	5.0 (± 3.3)	5.8 (± 3.4)	6.6 (± 3.2)
Enjoyment of life	8.5 (± 1.4)	6.8 (± 4.4)	6.7 (± 2.7)	8.0 (± 1.9)
Mean of all	7.6 (± 1.3)	6.5 (± 2.9)	6.9 (± 1.7)	7.1 (± 2.3)
States of anxiety and depression (HADS score, mean (±SD)):				
Anxiety	7.0 (± 3.7)	12.0 (± 4.5)	7.5 (± 4.4)	10.5 (± 5.3)
Depression	6.9 (± 0.7)	6.2 (± 1.3)	5.5 (± 2.2)	5.5 (± 3.1)
Neuropathic pain component (PD-Q score, mean (±SD))	9.0 (± 3.5)	9.6 (± 1.5)	9.6 (± 4.4)	8.8 (± 2.9)

^a^ “Normal” group includes patients with normal sensitivity in all tested modalities;

^b^ “1-3 hypo” group – patients with detected hyposensitivity in 1, 2, or 3 modalities;

^c^ “4-5 hypo” group – patients with detected hyposensitivity in 4 or 5 modalities;

^d^ “Hyper” group – patients with detected hypersensitivity in all tested modalities; LSR – lumbosacral radiculopathy; SD – standard deviation; BPI-SF – Brief Pain Inventory short form; NRS – numeric rating scale; HADS – Hospital Anxiety and Depression Scale; PD-Q – painDETECT Questionnaire.

The duration of the full testing battery was 29.3 ± 6.5 minutes per patient. Such duration seems quite adequate and corresponds to an intended 30 minutes for at least one test point of the standardized QST protocol by the German Research Network on Neuropathic Pain [14]. During this time, the applied testing permitted screening of multiple (L3-S2) segments and multiple test points along the whole segment length.

Exceptional attention was paid to patient selection (inclusion/exclusion criteria) to avoid any factors that might misrepresent sensory function and testing. Therefore, screening for patients with pure unilateral LSR and excluding any patients with diseases, conditions, or factors that could affect sensations in the areas of the body being studied was critical. A significant list of exclusion criteria considerably reduced the number of patients screened for inclusion, with 140 (68%) of 206 study candidates excluded. This is the primary limiting factor for this type of sensory testing to be used in clinical practice.

Also, good patient cooperation is essential to ensure the effective use of sensory testing. The patient cooperation level in our study was acceptable. The overall withdrawal rate due to inconsistent contact or linguistic issues was 8% (17 patients of 206).

All sensory testing instruments were easy to handle; no technical problems occurred. The price of the whole set of testing instruments was approximately 2000 euros. This is about 10 times less than comprehensive automated QST systems.

In our study, nine (18%) of the 50 subjects did not report any superficial disturbances (12%, if including deep tissue pressure evaluation). Normal sensations were found in 18% to 58% if the different tests were assessed separately. Intact sensory function or a relatively low degree of sensory impairment difficult to distinguish from the unaffected side of the body are among possible explanations. Qualitative type of sensory testing in patient populations with unilateral sciatica [15], in lumbar nerve-root compression syndromes [16], in radicular pain patients [17], or symptomatic lumbar lateral stenosis involving L5 root [9] showed similar frequencies (27% to 55%) of normal sensitivity.

The dominant impairment type detected in our study is hyposensitivity. This conforms with all other radiculopathy studies. Hyposensitivity to most superficial tests (to 4 or 5 tests) dominated across the study population and accounted for 31 (62%) of the 50 study patients.

Patients with hypersensitivity are another specific group. Five of the 50 patients showed hypersensitivity to all superficial tests. Another four patients showed isolated hypersensitivity to one modality (brush or cold perception), with hyposensitive responses to all other tests. While hypersensitivity phenomena were common in one report with qualitative type testing study [18], some other studies with QST report hypersensitivity as a rare finding [17, 19, 20]. Some other radiculopathy studies did not record hypersensitivity or included them in the general group of “sensory impairments ” [9]. Sensory hypersensitivity is a marker of nervous system sensitization [21]. Also, hypersensitivity could be related to sympathetic fibers sprouting on dorsal root ganglion cells [22].

Brush testing (200–400 mN) for tactile dynamic perception showed statistically significantly the smallest portion of hyposensitivity cases than other sensory modalities. This resembles findings of sensory testing with cotton swab stroke in atypical odontalgia patients [8]. 

Deep tissue sensitivity testing was performed with a nonstandardized instrument. The results of this test exhibited a relatively high percentage of hypersensitivity (44% in deep pressure test vs. 10–14% in other modalities) and the presence of mixed hypo- and hypersensitivity together in the same patient at different tested points (16% of patients). The most reliable explanation of such intraindividual mixed type disturbances could be the different underlying tissues (bony prominences, muscles with bony structures underneath at different depths) at various testing sites, which could be of great importance when testing the lower limb [23]. A mechanical pressure algometer was tested during study preparations as a possible instrument for deep tissue sensitivity testing. Maintenance of stable fixed pressure during the test appeared to be technically complex and/or inaccurate. It seemed confusing for patients to report perceived pressure since they had to evaluate only the final pressure achieved and maintained for several seconds. Therefore, finger pressure testing was chosen for testing, as it was recommended for bedside testing in some publications [17, 23]. To improve the set of bedside-suitable tests, a standardized deep tissue pressure testing instrument that would produce and maintain a fixed amount of pressure is needed. Also, a proper level of pressure that would be most informative for the screening of various body regions should be established.

The unexpected finding was the neuropathic scores obtained with PD-Q. In general, PD-Q scores in LSR group were low (9.5 ± 3.8) with 81% of patients qualified as ‘unlikely neuropathic pain’, 17% as ‘uncertain’, and only 2% as ‘likely neuropathic pain’. Given that study population consisted of carefully selected LSR patients, the results appear to be in line with publications on this subject [24].

We did not intend to measure the reliability of testing techniques. The use of nonstandardized testing instruments has already been investigated in atypical odontalgia patients [8] and exhibited sufficiently high intra-rater and inter-rater reliability. Also, some bedside sensory testing tools for detecting of cold, warm, mechanical, and pressure pain thresholds have already been proven to correlate with the respective QST parameters significantly [25]. We used strictly standardized instrumentality for superficial sensory testing. Therefore, it is likely that the reliability rates would not be lower than in the above study.

Data from QST studies usually report sensory function in group averages of quantitative indicators. However, individual variability remains unclear. Our study revealed considerable variability of sensory disturbances – nine sensory phenotypes were distinguished according to individual sensory profiles. This was also reported with QST in cervical radiculopathy [26]. The variety of such sensory phenotypes could reflect the diversity of causative spinal changes in our aged study population and various degrees of root impairment. Potentially, sensory testing data could be related to LSR treatment effects or other prognostic factors. The available data are contradicting. The current perception threshold testing was evaluated in patients with lumbar radiculopathy before and after epidural steroid injection [27] without correlation in pain intensity changes after the treatment. Later studies with QST showed some potential to predict improvement after epidural steroid injection for sciatica-related pain [28] and in predicting functional status after microdiscectomy in patients with LSR [20]. QST could potentially reveal the treatment implicated mechanism of action of spinal manipulative therapy [29]. A review and meta-analysis of QST related prediction of musculoskeletal pain-related outcomes state that QST might help to identify persons to benefit most from treatment interventions [30]. Further studies are needed to address the optimal bedside testing battery issue to detect sensory changes determinant for decision-making.

## Conclusion

The applied multimodal bedside-suitable somatosensory testing battery is suitable for sensory evaluation, characterization and phenotyping of LSR patients with radicular pain. Tactile static testing with a 10g monofilament showed the highest sensitivity to detect superficial sensory disturbances. It could be recommended as the most sensitive single testing instrument. Tactile dynamic testing with 200–400 mN brush detected sensory impairments significantly less frequently than any other modalities, therefore not recommended for the screening of LSR patients. The detected sensory phenotypes should be explored for the relation with treatment effects.
